# Surgical Removal of a Long-standing Impacted Tooth in Upper Alveolus Following Blast Injury: A Case Report

**DOI:** 10.31729/jnma.8864

**Published:** 2025-01-31

**Authors:** Arun Adhikari, Shila Acharya, Sadhana Sharma, Luna Mathema, Bijay Khatri

**Affiliations:** 1 Department of Otolaryngology & HNS, B.P. Eye Foundation, Hospital for Children, Eye, ENT, and Kehabilitation Services, Madhyapur Thimi, Bhaktapur, Nepal; 2 Academic and Research Department, B.P. Eye Foundation, Hospital for Children, Eye, ENT, and Kehabilitation Services, Madhyapur Thimi, BhakTapur, Nepal

**Keywords:** *blast injury*, *computed tomography*, *foreign body*

## Abstract

Injuries sustained from foreign bodies and burns after oil drum explosions are addressed immediately. This case reports a rare long-standing retained tooth after such an explosion. A 65-year-old male with a history of a victim of explosion injury to the face dating 20 years back with complaints of repeated nasal vestibulitis was evaluated. An X-ray of paranasal sinus followed by computed tomography of the nose and paranasal sinuses revealed a radio-opaque foreign body in the region of upper alveolus near left nasal vestibule. After consultation and clearance from dental department, exploration through the vestibule was done and the foreign body was removed without complications. Our intervention involved surgical removal of foreign body under local anesthesia. The patient has recovered following the intervention and has resumed his normal activities.

## INTRODUCTION

The nature of injuries resulting from explosions varies based on factors such as materials involved, environment, victim's proximity to explosion, and presence of protective barriers.^1^ Oil drums containing various inflammable liquids, if ignited, can cause an explosion causing extensive injuries or deaths. ^2,3^ Besides fragments from such vessels become missiles which can cause penetrating and damaging wounds. Most injuries are managed immediately by removing foreign body and treating burns. However, in some cases, foreign bodies are missed and remain embedded in soft tissues. It can lead to infection often, requiring multiple medical attention. This case reports surgical management of a foreign body stuck in upper alveolus for 20 years, which was missed on multiple consultation.

## CASE REPORT

A 65-year-old male, presented himself at Ear Nose Throat (ENT) Out Patient Department (OPD) of our hospital, with complaints of infection of the left nasal vestibule on and off for the last 15 years. He reported that he was a victim of a petrol drum explosion 20 years back as a foreign employee in Dubai, United Arab Emirates. He was injured in 2002 while working at a petrol station. Immediately after, he was taken to the nearby hospital, where he found that his upper incisors and both canine teeth were absent along with the surrounding injury. He was assessed and surgery was performed to remove the buried teeth. After the incident, he did not have any issues for around 5 years. However, he started developing repeated vestibulitis and was prescribed oral and topical antibiotics.

On initial inspection and physical examination at OPD, there was a stony hard foreign body at the left nasal vestibule with an overlying ulcer, the foreign body was not adhered to the underlying structure and was adhered to the overlying skin. The margin of the ulcer was well-epithelized.

**Figure 1:**
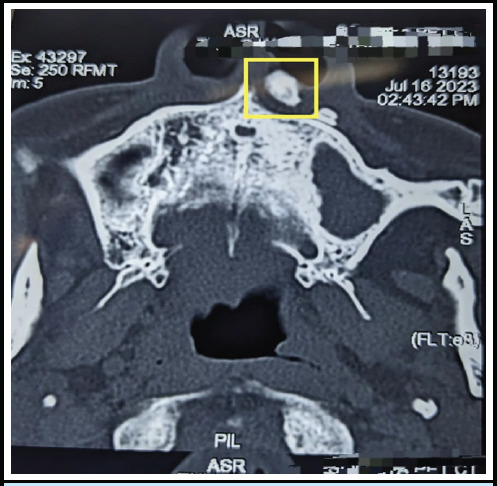
CT scan showing hyperdense focus in the left premaxillary soft tissue plane (inside yellow highlighted box).

Oral examination and anterior rhinoscopy were performed which showed no other abnormality. X-ray Paranasal sinus occipitomental view showed radioopaque foreign body at upper alveolus. A computed tomography scan of the nose and paranasal sinus showed hyperdense focus in the left premaxillary soft tissue plane, attenuation of which is similar to the maxillary tooth without a definite soft tissue component. It lied just below the skin of the left nasal vestibule and it was not in continuation to the upper incisors (Figure 1).

Dental consultation was sought for the clearance of surgical removal and further assistance during surgery, if required. The patient had no history of drug allergies. As the patient had no added comorbidities and any other health related issues requiring general anesthesia, written consent from the patient and patient party were taken and exploration and foreign body removal under local anesthesia was planned. The pre-operative investigations of blood showed he was fit for surgery under local anesthesia.

**Figure 2:**
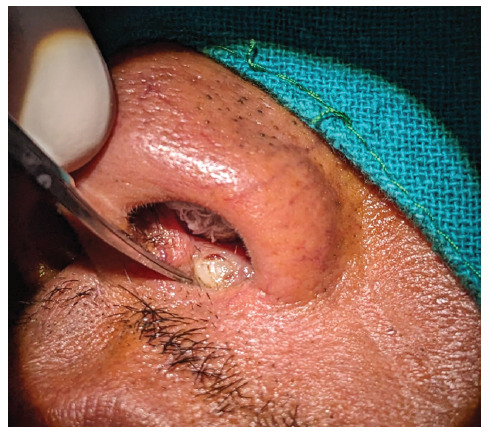
Anterior nasal packing with ribbon gauge and foreign body seen in the left nasal vestibular area.

**Figure 3:**
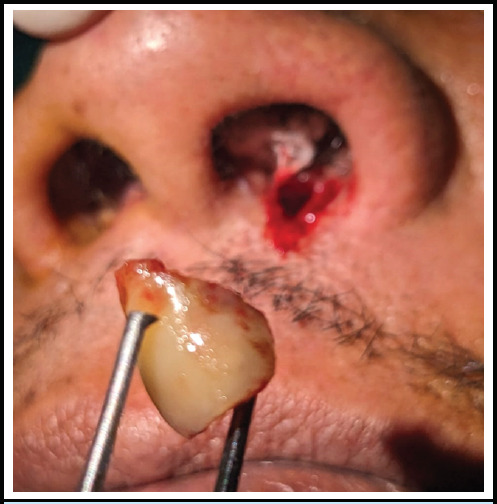
Foreign body just after removal.

**Figure 4:**
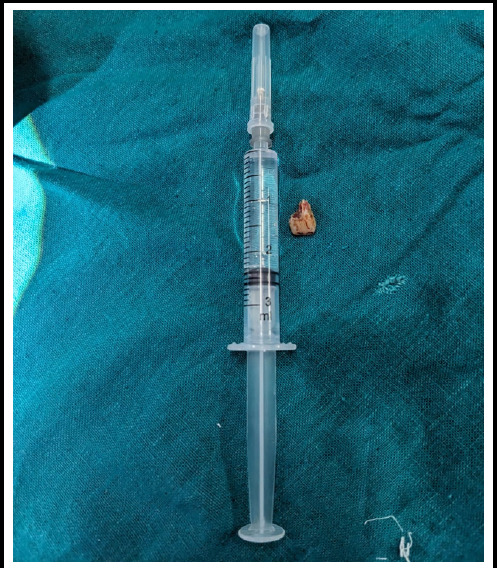
Removed foreign body compared to a 3ml syringe (tooth).

Under local anesthesia, the patient was placed in the supine position with all aseptic precautions. Left nose was packed with normal saline soaked ribbon gauze to prevent blood from reaching his throat. (Figure 2). A horizontal incision was given using no.15 surgical blade along the foreign body at the nasal vestibule and dissected till the surrounding fibrosed tissue. The fibrosed tissue was dissected using plane scissors and the foreign body was removed with the help of toothed forceps which is found to be the part of the tooth (Figure 3 and 4). After removal, a potential space was present which was not communicating with the oral cavity. The wound was closed in two layers, inner layer by vicryl 4.0 and outer layer by 4.0 prolene. The intervention lasted for around 30 minutes.

We did not have any complications or unanticipated events to report during the surgery. The patient was discharged on the same day and was advised to take antibiotics, painkillers, and anti-inflammatory drugs for a week, and sutures were removed on the seventh post-operative day. He was advised to take painkillers if required and asked to visit after a week following the suture removal. A fortnight after the surgery, the patient reported no apposition of the incision site. Hence, artificial collagen particle along with antibiotic ointment was used every 72 hours at home by the patient himself.

Four weeks following surgery, the patient reported resuming his daily chores and his wound was apposed completely during the follow-up visit. Ten weeks after surgery, the patient is doing fine and has resumed his daily activities.

## DISCUSSION

There are four types of blast injuries. Primary blast injuries are caused by the direct impact of the pressure wave on the body surface, organs with air-fluid interfaces are particularly susceptible to injury. A secondary blast injury results from flying debris and can cause both blunt and penetrating injuries. Tertiary blast injury occurs when victims are thrown against solid objects by the blast wave. The quaternary blast injury encompasses miscellaneous injuries such as burns and crush injuries and illnesses directly related to the blast such as post-traumatic stress disorder, bronchospasm, angina, or inhalation of toxic fumes.^1^

Secondary blast injury is the most common type of injury associated with explosive blast incidents.^1^ Our case also falls into secondary blast injuries as the impact to tooth projected it into the upper lip and eventually into the left vestibule of nose. Injuries to the head, face, and neck vary greatly, spanning from minor incidents not necessitating surgical intervention to those posing serious, life-threatening risks.^4^

A mass casualty incident can overwhelm local hospital resources.^5^ Based on on-site and emergency triage, this patient may have got little attention and treated for minor injuries and burns and visible impacted teeth removed. Further examination and medical experts' reassessment of injury severity is of paramount importance following primary treatment, as required. But, due to the language barrier, and high cost of treatment for labor migrants in gulf countries, he halted the follow-up process. This resulted in impacted foreign body in the upper alveolus for a long time and repeated complaints of nasal vestibulitis. The tooth, as foreign body embedded in the floor of the upper alveolus for more than 20 years is quite rare.

The patient claimed a petrol drum blast in this case. However, as the vital structures were not involved and he was able to contain the foreign body (tooth) for more than 20 years, it is possible that the tooth struck just above the gums and then appeared in the left nasal vestibule with decreased velocity. The nasal crust was visible just above the upper lip in the left vestibular region, where the foreign body was palpable and was impacted within the soft tissue.

The X-ray helped to delineate a radio-opaque foreign body at the upper alveolus. Computed Tomography scan helped us to differentiate whether the foreign body is adhered to the other body structures. It also helped to visualize if there are other foreign bodies present so that surgery can be planned accordingly. A case study has suggested that for patients presenting with recurrent symptoms in ENT, especially after traumatic accident, maintaining a suspicion of a foreign body is essential and CT scan should be done to explore this possibility.^6^ Computed tomography scan also had earlier helped removal of 16 years long-standing impacted firearm from neck.^7^ Computed tomography scans have been found useful in extraction of wooden or metallic foreign bodies.^8,9^ Computed tomography scan is the preferred examination for locating a foreign body and evaluating its relationship with surrounding structures to facilitate possible extraction.^8^ It plays a crucial role in assessing the foreign body in relation to adjacent tissues.^9^ Clinicians should be aware that multiple mechanisms are effective, based on location of foreign bodies especially in case of history of injuries and trauma, and therefore a detailed radiological examination should be undertaken. While MRI avoids radiation, caution is needed if the foreign body may have a magnetic component.

A long-standing foreign body can lead to non-healing wounds^10^ and repeated infections, as in our case. It can also cause fibrosis of the surrounding tissues, making future removal more complicated. Additionally, a longstanding foreign body may lead to chronic neuralgic pain by irritating nearby nerves. ^7^

The patient is satisfied post-operatively as he no longer requires frequent visit to the hospital for his nasal vestibulitis. This case illustrates that the patient complaints should be of utmost importance and referring the patient to specialized surgeons and each of these types of injuries are unique to each other.

The presentation of the impacted foreign body resulting from blast injury may vary depending on the location within the human body. The preferred treatment will be determined by the clinical presentation of each case, as well as the diagnostic accuracy and timely management. This approach can alleviate the pain, as well the psychological and financial burdens associated with such injuries.

## References

[1] Juan A, Trunkey DD, Asensio (2016). Current Therapy of Trauma and Surgical Critical Care..

[2] Back B, Juhl M, Lauridsen F, Pilegaard J, Roeck ND (1988). Oil and Petrol Drum Explosions. Injuries and Casualties by Exploding Oil and Petrol Drums Containing Various Inflammable Liquids.. Injury..

[3] Kumar P (2013). Fire Disaster Following LPG Tanker Explosion At Chala In Kannur (Kerala, India): August 27, 2012.. Burns..

[4] Tahtabasi M, Er S, Kalayci M (2020). Head And Neck Injuries After A Bomb Explosion: Diagnostic Findings And Treatment Approaches.. Am J Otolaryngol..

[5] Ahmad S (2018). Mass Casualty Incident Management.. Mo Med..

[6] Bazzout A, Lachkar A, El Ayoubi F, Abdenbi Tsen A, Ghailan R (2021). A Foreign Body Lodged In The Ethmoid Sinus 3 Years Previously: A Case Report.. Int J Surg Case Rep..

[7] Adhikari A, Mahoto NB, Khatri B (2022). Surgical Removal of a Long-Standing Impacted Firearm In Neck: A Case Report.. JNMA J Nepal Med Assoc..

[8] Mori S, Fujieda S, Tanaka T, Saito H (1999). Numerous Transorbital Wooden Foreign Bodies in the Sphenoid Sinus.. ORL J Otorhinolaryngol Relat Spec..

[9] Padiyar BV, Vats A, Dhiman A, Rai AK (2019). Impacted Bullet in the Sphenoid Sinus: A Case Report.. Dubai Medical Journal..

[10] Thakur A, Sharma A (2024). Chronic Nonhealing Wound Due to Iatrogenic Foreign Body- A Case Report.. J Indian Assoc Pediatr Surg..

